# Patient communication pattern scale: psychometric characteristics

**DOI:** 10.1111/hex.12381

**Published:** 2015-07-14

**Authors:** Sara Ilan, Sara Carmel

**Affiliations:** ^1^ Department of Public HealthFaculty of Health SciencesBen‐Gurion University of the NegevBeer‐ShevaIsrael; ^2^ Head Center for Multidisciplinary Research in AgingBen‐Gurion University of the NegevBeer‐ShevaIsrael

**Keywords:** communication patterns, doctor–patient relations, shared decision making

## Abstract

**Background:**

In western societies, a shared decision‐making model for doctor–patient relationships calling for open and collaborative communication is recommended. Research focuses mainly on the doctor's communication patterns, while research on patient communication patterns is rare. The purpose of this study was to develop a tool for evaluating patient's communication patterns – the Patient Communication Pattern Scale (PCPS).

**Methods:**

Interviews based on structured questionnaires were conducted with 251 cancer patients. In addition to the 14‐item PCPS, the questionnaire included questions regarding education, religiosity and desirability of control in general and over one's own health in particular, for validating the scale.

**Results:**

The PCPS was found to be a valid and reliable tool for evaluating patients' communication patterns. Confirmatory factor analysis supported the PCPS designed structure of five facets: (1) Information, (2) Clarification, (3) Initiation, (4) Preferences and (5) Emotions.

**Conclusion:**

The PCPS is a reliable scale for evaluating patient communication patterns. The use of this scale can assist in promoting related research and in developing interventions for enhancing open and collaborative doctor–patient communication.

## Introduction

In recent decades, significant changes have occurred in approaches concerning the doctor–patient relationship. This relationship has evolved from the paternalistic model dominant in the fifties and sixties of the last century,^1^ through the informative model (or customer model) prevailing in some countries in the seventies,^2^ to the modern collaborative model of doctor–patient relationship that acknowledges a patient's right to fully participate in the medical decision process. These three basic models of doctor–patient relationship – paternalistic, informative and collaborative – differ in the perception of physician and patient roles, patient autonomy and the importance of the patient's values and preferences in the decision‐making process.^3^, ^4^ Unlike the paternalistic relationship model, which assumes that the physician knows what is best for the patient, or the informative model, in which the decision‐making process is transferred to the patient,^3^ in the collaborative model, both doctor and patient are involved in the decision‐making process, and considered equal partners.

This conceptual transformation has been endorsed by many countries (including Israel) and is supported by legislation on patient's rights.^5^, ^6^ However, many difficulties are reported in the implementation of this model: partial disclosure of information (on both sides)^7^, ^8^, ^9^, ^10^, ^11^, inadequate provision of information^12^, ^13^, interrupting patient^14^, ^15^, difficulties in addressing patient's feelings and worries^15^, ^16^, ^17^, ^18^, discussing patient's preferences^19^, ^20^ and collaborative decision making^19^, ^21^, ^22^, ^23^. Predominant difficulties in open and collaborative communication between the parties especially exist when the patients suffer from severe and terminal diseases,^24^, ^25^, ^26^, ^27^, ^28^, ^29^, ^30^ although the importance of open and collaborative communication in such conditions is greater.^17^, ^31^, ^32^, ^33^ In an effort to understand this situation, research has focused on the physician's communication patterns while studies on the patient's role in this dyad are rare.^34^, ^35^ The purpose of this study was to develop and validate a scale for evaluating the patient's communication patterns.

### Doctor–patient communication in the collaborative model

Doctor–patient communication is a multidimensional process, which on the doctor's part includes delivering clear and complete information about the disease, its symptoms, possible treatments, their side‐effects and the prognosis.^7^, ^36^, ^37^, ^38^ In addition, doctor's assessment of the patient's understanding of the information is recommended,^27^, ^39^ as well as discussing patient's willingness to be involved in the decision‐making process,^26^, ^40^ and support the patient emotionally.^41^, ^42^, ^43^ Similarly, the patient is expected to provide complete information regarding his/her symptoms,^40^, ^44^, ^45^, ^46^, ^47^ ask questions regarding unclear information^45^, ^46^, ^47^ and/or lacking information such as medical treatments and procedures, side‐effects and prognosis.^40^, ^45^, ^48^ In addition, expressing fears and worries by the patient is advisable.^18^, ^41^, ^49^ In this model of interaction, the physician's knowledge of the patients' preferences and willingness to take part in the medical decision‐making process are of special importance.^26^, ^46^, ^50^

Several measures have been developed to evaluate physician communication patterns.^45^, ^51^, ^52^, ^53^, ^54^ As to the patients' communication behaviour, Galassi *et al*.^45^ developed the Patient Reactions Assessment (PRA) which includes three subscales, two of them for evaluating the medical provider's contribution to the relationship, while the third subscale – the Patient Communication Index (PCI) – was developed to assess only the patient's ability to initiate communication about his/her illness. Lerman *et al*.^46^ developed the Patients' Perceived Involvement in Care Scale, which also includes three subscales: one measures physician behaviour, while the Patient Information Scale contains statements regarding patient level of information providing, symptoms and medical recommendation, and the Patient Decision‐Making Scale which includes statements regarding the patient's suggestions and agreement to medical tests and treatment. However, certain aspects of care and patient communication, such as side‐effects, prognosis, quality of life, fears and other‐related emotions, are lacking in the previously developed scales. These aspects, which are important for evaluating the patient's behaviour in the physician–patient interaction, have been included in the PCPS.

### Patient Communication Pattern Scale (PCPS)

Considering the changes in the patient's rights and role in the doctor–patient interaction, and the absence of a complete measure to evaluate patient communication patterns, we developed the PCPS based on the above‐mentioned communication literature, a qualitative study conducted on 12 oncologists in a number of medical centres in central Israel to understand how they perceive patients' preferences and behaviour in the doctor–patient interactions. The qualitative study supports the literature and suggests that only partial information is provided to patients, especially with respect to treatment options, side‐effects and possibilities of recovery. The oncologists claim that patients primarily ask questions about side‐effects and do not ask about prognosis; patient's preferences are hardly discussed and the treatments fit physician recommendations based on their experience and preferences rather than those of the patient. Conceptually, the PCPS was designed to encompass five different facets of communication: (1) relaying clear information about the illness and symptoms; (2) questioning and requesting clarifications; (3) initiating request for information from the physician; (4) guiding the physician according to one's own preferences; (5) reporting one's feelings.

#### Scale validation

Age, education, religiosity and desirability of control (in general and over the patient's own health in particular) were used to validate the scale. The literature indicates that elderly patients are more passive in doctor–patient interactions than younger patients,^49^, ^55^, ^56^ tend to ask less questions,^47^, ^48^ do not present their feelings as much as others^47^ and are more likely to leave the treatment decisions to their physician.^57^, ^58^ Studies also show that educated patients tend to ask more questions than less educated patients^48^ and are more willing to participate in decision‐making processes concerning their health.^58^ Religiosity was also found to be related to the level of patients' preference for a collaborative model of doctor–patient interaction. For example, religious Israeli Jewish patients wanted less open communication with their physician about end‐of‐life care than secular patients,^59^ and American religious individuals had higher levels of trust in their physicians than the less religious.^60^ Consequently, we hypothesized that patients' age and religiosity would be negatively associated with patient's scores on the PCPS, while patients' education would positively correlate with their PCPS scores. Desirability of control reflects the degree to which a person is motivated to control his/her life's events.^61^ Desirability of control (in general and over the patient's own health in particular) was assumed to positively correlate with patients' PCPS scores, as seeking as much information as possible is often the way of people to cope with uncertainty and gain more control over their lives.^62^, ^63^

## Methods

### Participants and procedure

The PCPS statements were formulated based on the literature, a qualitative study with oncologists, and a pre‐test conducted on 25 patients waiting for appointments to their family physicians in community clinics. The interviews were approved by the Helsinki Committees of the Clalit Health Services (the largest HMO in Israel). The study for testing the PCPS was performed in an oncology day‐care clinic of one of the largest medical centres in Israel that receives about 3000 new patients every year. Study procedures were approved by the Helsinki Committees of this medical centre which belongs to the Clalit Health Services. Patients who visited the oncology clinic for consultation and/or treatment and were Hebrew speaking and cognitively competent and able to independently answer questions, were asked to participate in the study. Those who agreed to participate in the study signed consent forms. Altogether, 251 adult patients with cancer participated in the study, 140 women and 111 men. The average age of the participants was 60.7 (SD = 3.1) ranging from 22 to 88 years; 247 (98.4%) of the patients were Jewish, 78.1% were married and 35.5% had an academic degree (Table 1).

**Table 1:** Socio‐demographic characteristics (*n* = 251)

Variable	No. of patients (per cent)
Gender	
Men	111 (44.2)
Women	140 (55.8)
Age	
Up to 30	7 (2.8)
31–44	28 (11.2)
45–64	113 (45.0)
65+	103 (41.0)
Range: 22–88	
Average age: 60.7 (SD = 13.8)	
Family Status	
Living with spouse	196 (78.1)
Single	17 (6.8)
Widowed	24 (9.5)
Divorced	14 (5.6)
Education	
Up to 8 years	19 (7.6)
Partial High School	12 (4.8)
High School	86 (34.3)
Tertiary	45 (17.9)
Academic	89 (35.4)
Religiosity^a^	
Non‐religious	147 (59.5)
Traditional	52 (21.1)
Religious	26 (10.5)
Orthodox	10 (4.0)
Ultra‐Orthodox	12 (4.9)

a Religiosity refers to Jewish patients only (*N* = 247).

### Measures

#### Patient Communication Pattern Scale (PCPS)

The PCPS was developed based on a literature review of the dimensions of communication, a qualitative study with oncologists and a pre‐test on 25 patients. A factor analysis conducted on the responses of the 251 participants to 16 statements yielded five different facets of communication: (1) Relaying clear information about the illness and symptoms (Information) – evaluated by two statements: ‘I gave the doctor complete information about the physical symptoms I suffer from’, and ‘I explained my problems to the doctor in a direct and clear way’; (2) Questioning and requesting clarifications (Clarification) – assessed by two statements: ‘When something in our conversations was unclear to me I asked the doctor to explain to me’, and ‘During the conversations I asked the doctor questions’; (3) initiating request for of information from the physician (Initiation) – evaluated by four statements: ‘I did not ask the doctor about my chances for my recovery and how much time I have left to live’, ‘I asked the doctor what are all the possible treatments for my condition’, ‘I asked the doctor how each treatment is carried out and what its side‐effects are’, and ‘I asked the doctor what the chances for my recovery are for each of the possible treatments’; (4) Guiding the physician according to one's own preferences (Preferences) – four statements: ‘In our conversations I was the one who initiated reference to my preferences for treatment’, ‘I made it clear to the doctor what is more important to me – quality of life or extended life’, ‘I made it clear to the doctor what is most important to me now as a result of my illness’, and ‘I was a full partner in making the decision about which treatment to choose’; (5) Reporting one's own feelings (Emotions) – two statements, ‘I discussed my fears and worries with the doctor’, and ‘In our conversations I was the one who initiated reference to my emotions’. The results of the factor analysis lead us to leave out two statements from the questionnaire: ‘I made it clear to the physician to whom of my relatives he can deliver information regarding my illness’ and ‘I showed respect to my physician’; the first statement had a low loading, and the second reduced the internal consistency of the factor it belong to.

The final questionnaire consisted of 14 (of the original 16) statements that characterize patient communication patterns (the questionnaire was generated and used in the Hebrew Language, and later translated to English, using the back translation technique, see Appendix A). The patient had to express agreement on a 6‐point scale, ranging from 1 – strongly disagree to 6 – strongly agree. The final general PCPS score and the scores on each of the 5 facets were indices built upon the average scores of responses given for the relevant items (after reversing item 10). Higher score represents a more open and collaborative communication pattern.

#### Age, education, religiosity and desirability of control

Age – patients were asked to respond in what year they were born, and we calculated their age accordingly. Education was assessed by responses to being told to choose one of five levels of education: up to 8 years, partial high school, high school, tertiary and academic studies (see Table 1). Religiosity was measured by a short version of the Jewish Religiosity Scale; the score is the sum of five items: strength of belief, self‐perceived religiosity, level of religiosity while patient was growing up, attending religious ceremonies and adherence to Jewish dietary laws,^64^ with higher scores presenting higher levels of religiosity. General desirability of control was assessed by a 13‐item scale ( Appendix B), which is a shortened version of the Desirability of Control Scale developed by Burger and Cooper^61^. Desirability of control over the patient's own health was measured by one item: ‘Regarding health matters, I've always preferred that the doctor decides what is best for me.”; the patient was asked to express agreement on a 6‐point scale, ranging from 1 – completely incorrect to 6 – completely correct (the answers were reversed, so that a higher score indicates a higher desirability of control over the patient's own health).

### Statistical analysis

After conducting a factor analysis which revealed 5 facets, the structure of the PCPS was evaluated using confirmatory factor analysis (CFA), with AMOS 18.0. To validate the PCPS, we used Pearson correlation coefficients for evaluating the associations between PCPS and age, religiosity and desirability of control, and Spearman correlation coefficients for evaluating the correlations between PCPS and education.

## Results

### The PCPS

Average scores and internal consistency of the indices are presented in Table 2. The factor analysis resulted in five factors. The general PCPS scores of our participants were relatively low with an average of 3.05 (SD = 0.85). When referring to the different facets of communication (Table 3), the highest scores were given to provision of information (Mean = 5.49, SD = 1.34) and to questioning and requesting clarifications from the physicians (Mean = 5.10, SD = 1.46). The lowest scores were given to reporting one's own feelings (Mean = 1.61, SD = 1.39), and guiding the physician according to one's preferences (Mean = 1.69, SD = 1.06). Internal reliability of the general index and each of the 5 indices were assessed by Cronbach's alpha and Pearson correlation coefficients. The reliability of the general index which was comprised of all the statements was moderate (α = 0.78). The alphas for two indices, comprised of four items each (Preferences and Initiation), were lower (α = 0.67 and α = 0.69, respectively), and the correlations for three of the indices, comprised of two statements each (Information, Clarification and Emotions), ranged from *r* = 0.65 to *r* = 0.95.

**Table 2:** Description of the Patient Communication Pattern Scale (PCPS) indices

Scale	No. of items	Mean (SD)	Internal Consistency
PCPS	14	3.05 (0.85)	α = 0.78
Providing information	2	5.49 (1.34)	*r* = 0.95
Questioning, requesting clarification	2	5.10 (1.46)	*r* = 0.65
Initiating requests for information	4	2.75 (1.50)	α = 0.69
Reporting Preferences	4	1.69 (1.06)	α = 0.67
Reporting emotions	2	1.61 (1.39)	*r* = 0.90

**Table 3:** Correlations between the different facets of communication

	Information	Clarification	Initiation	Preferences	Emotions
Information	1				
Clarification	0.161*	1			
Initiation	0.071	0.379**	1		
Preferences	0.036	0.235**	0.437**	1	
Emotions	0.127*	0.175**	0.204**	0.327**	1

*p < 0.05, **p < 0.01

The highest correlations were found between Preferences and Initiation (Table 3) (*r* = 0.44) and between Initiation and Clarification (*r* = 0.38).

#### Confirmatory Factor Analysis (CFA)

CFA was used to confirm an *a priori* hypothesis regarding items which should be grouped together.^65^ The chi‐squared, comparative fit indices (CFI), root mean square error of approximation (RMSEA) and HOELTER were used for evaluating and comparing models across the CFA.^66^, ^67^

**Figure 1:**
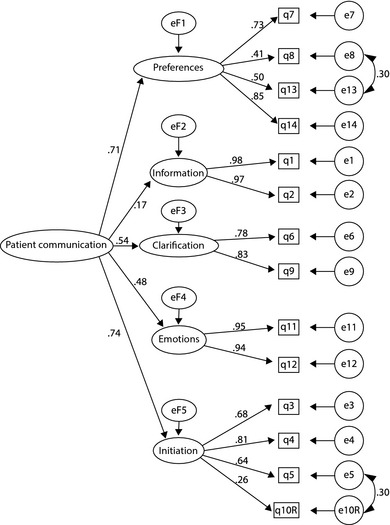
Results of a confirmatory factor analysis on the items of the PCPS.

After excluding all missing cases, responses of 223 patients were examined by CFA. In the first CFA model, the Information factor's loading was very low (0.16), but theoretically ‘relaying clear information about the illness and symptoms’ is part of the communication behaviour of the patient; additionally, taking out this factor worsened all the fit indices. The recommendation of the modifications in CFA included correlations between statements 8 and 13, and 5 and 10 (reversed); adding these correlations (one by one) to the CFA model improved all of the indices, but at the same time, adding the correlation between 5 and 10 (reversed) reduced the loading of item 10 (reversed) to 0.26. The fit indices for the final model were: χ^2^/d.f. = 1.265 (not significant); CFI = 0.988; RMSEA = 0.035; HOELTER = 227 (*P* = 0.05). All of these indices indicate a good model fit to the data (Fig. 1).

Validity was assessed by correlations between the PCPS and age, education, religiosity and desirability of control in general and over the patient's own health. Although all of these associations were found to be statistically significant, some of them were relatively low. Therefore, we conducted a linear multiple regression analysis on PCPS as a dependent variable and all of these variables as independent variables. This model was found to be statistically significant, explaining 19% of the variability in PCPS (adjusted *R*² = 0.188). In this analysis, only age (B = −0.018, β = −0.295, *t* = −4.842, *P* < 0.001), education (B = 0.169, β = −0.2395, *t* = 3.909, *P* < 0.001) and desirability of control in general (B = 0.172, β = 0.124, *t* = 2.63, *P* = 0.04) remained statistically significant explanatory variables of PCPS.

## Discussion

The purpose of this study was to enhance research on patient communication patterns by developing the PCPS and testing both its reliability and validity. This scale was developed in response to current social developments regarding the preferred model of doctor–patient relationship in Western societies and the related research. The current dominant approach in these countries emphasizes patient autonomy including active expression of personal values and preferences in the doctor–patient relationship. Ensuring these aspects in the medical decision‐making process requires open and collaborative communication between both parties – doctor and patient. Open communication includes discussion about all aspects of the disease, treatment, prognosis, patient preferences and feelings. Considering the dominant role of doctors in the previously prevailing paternalistic model of the doctor–patient relationship, these social developments naturally lead to studies on physicians' adaptation to the new requirements. This body of research includes studies on doctor communication patterns within the doctor–patient relationship, while research on patient communication patterns has been quite rare.

The PCPS described in this study was developed based on a literature review, a qualitative study among oncologists who were asked to describe their patients' communication patterns, and a pre‐test conducted on Israeli patients with cancer. The psychometric characteristics of the scale were assessed in a study conducted on 251 patients with cancer. Our findings support the *a priori* 5‐facet conceptual structure of the PCPS. All of the indices we used indicated good fit of the model to the collected data.

Regarding the validity of the PCPS, as exp‐ected, univariate analyses resulted in statistically significant negative correlations between PCPS scores and both age and religiosity, and significant positive correlations between the PCPS scores and education and desirability of control scores (both general and over the patient's own health in particular). However, only age, education and desirability of control in general were found to be significantly explanatory factors of the variability in PCPS in the expected directions. In addition to supporting the validity of the scale, these findings indicate that the younger patients are and the higher is their education and need for control, the more they use open and collaborative communication patterns in doctor–patient interactions.

As regard to patient communication patterns, our findings indicate partial openness and collaboration among Israeli patients with cancer. It appears that patients relay information about their physical symptoms and ask questions about the information that is provided, but almost never request any further information. Patients rarely ask about other or additional possible treatments, side‐effects or prognosis for recovery. Generally, patients also avoid initiating discussion about personal preferences and feelings.

When comparing the results of this study to previous reports, our findings also indicate a significant discrepancy between patient desires and behaviour. Generally, when asked, patients express a desire to receive as much information as possible about their diseases,^57^, ^68^, ^69^, ^70^ and to take part in treatment decisions.^40^, ^58^, ^71^ However, there is hardly any evidence that these desires are acted upon in their behaviours within the patient–physician interaction, in support of previous findings of studies conducted on doctors and patients in Israel and other countries.^22^, ^24^, ^47^, ^49^

Our findings should be viewed in the light of the limitations of the current study: the internal consistency of two of our subscales – initiating request for information from the physician (Initiation) and guiding the physician according to one's own preferences (Preferences) – is low to moderate. Also, the general scale includes only one statement that is negatively phrased; this item has a low loading in the final CFA, which might be explained by its' negative wording. In addition, this study was conducted on patients with cancer in Israel. Therefore, further research to be conducted in other countries and on patients with other diseases is needed in order to broaden our understanding of patients' communication patterns.

Aside from its limitations, this study leads to the conclusion that in spite of current recommendations for open communication and active patient participation in the medical decision process,^41^, ^72^ it seems that patients have difficulties in exercising their wishes and rights, or may not be willing to know more about their diseases, especially when suffering from severe diseases such as cancer. To promote the collaborative model of doctor–patient interaction, it is desirable that physician enhance their communication skills, including techniques for eliciting patient preferences and encouraging participation in the decision‐making process.

Regarding the PCPS, it was found to be a valid and useful tool for evaluating and understanding patient communication patterns, and for identifying the facets, which are not openly addressed. We are becoming more aware of the importance of open and collaborative doctor–patient communication, especially when patients suffer from severe and/or terminal diseases. From a practical point of view, the PCPS can be used for evaluating the level of patients' communication pattern on each of the scale's five dimensions. Such evaluations can lead further to the development of effective interventions for empowering patients to become more active participants in the medical decision‐making process.

## Funding

No source of funds.

## Appendix A

### Patient Communication Pattern Scale (PCPS)

The following items refer to your meetings with the doctor who treats you regularly.

Please rank your degree of agreement for each of the items on the scale ranging from 1 = strongly disagree to 6 = strongly agree.

**Table:**

Statements	Strongly disagree	Disagree	Somewhat disagree	Somewhat agree	Agree	Strongly agree
1. I gave the doctor complete information about the physical symptoms I suffer from	1	2	3	4	5	6
2. I explained my problems to the doctor in a direct and clear way	1	2	3	4	5	6
3. I asked the doctor what are all the possible treatments for my condition	1	2	3	4	5	6
4. I asked the doctor how each treatment is carried out and what its side effects are	1	2	3	4	5	6
5. I asked the doctor what the chances for my recovery are for each of the possible treatments	1	2	3	4	5	6
6. When something in our conversations was unclear to me, I asked the doctor to explain it	1	2	3	4	5	6
7. I made it clear to the doctor what is more important to me – quality of life or extended life	1	2	3	4	5	6
8. I was a full partner in making the decision which treatment to choose	1	2	3	4	5	6
9. During the conversations I asked the doctor questions	1	2	3	4	5	6
10. I did not ask the doctor what my chances for my recovery are and how much time I have left to live	1	2	3	4	5	6
11. I discussed my fears and worries with the doctor	1	2	3	4	5	6
12. In our conversations, I was the one who initiated reference to my emotions	1	2	3	4	5	6
13. In our conversations, I was the one who initiated reference to my preferences for treatment	1	2	3	4	5	6
14. I made it clear to the doctor what is most important to me now as a result of my illness	1	2	3	4	5	6

## Appendix B

### Desirability of Control Scale (a short version of Burger & Cooper's Scale)

Please rank your degree of agreement for each of the items on the scale ranging from 1 = completely incorrect to 6 = completely correct.

**Table:**

Statements	Completely incorrect	Incorrect	Somewhat incorrect	Somewhat correct	Correct	Completely correct
1. I prefer a job where I have a lot of control over what I do and when I do it	1	2	3	4	5	6
2. I try to avoid situations where someone else tells me what to do	1	2	3	4	5	6
3. I enjoy being able to influence the actions of others	1	2	3	4	5	6
4. Others usually know what is best for me	1	2	3	4	5	6
5. I enjoy making my own decisions	1	2	3	4	5	6
6. I enjoy having control over my own destiny	1	2	3	4	5	6
7. I consider myself to be generally more capable of handling situations than others are	1	2	3	4	5	6
8. I'd rather run my own business and make my own mistakes than listen to someone else's orders	1	2	3	4	5	6
9. When I see a problem I prefer to do something about it rather than sit by and let it continue	1	2	3	4	5	6
10. When it comes to orders, I would rather give them than receive them	1	2	3	4	5	6
11. I wish I could push many of life's daily decisions off on someone else	1	2	3	4	5	6
12. I prefer to avoid situations where someone else has to tell me what it is I should be doing	1	2	3	4	5	6
13. I like to wait and see if someone else is going to solve a problem so that I don't have to be bothered by it	1	2	3	4	5	6

## References

[1] Parsons T . Suggestions for a sociological approach to the theory of organizations‐I. Administrative Science Quarterly, 1956; 1: 63–85.

[2] Katz E , Gurevitch M , Peled T , Danet B . Doctor‐patient exchanges: a diagnostic approach to organizations and professions. Human Relations, 1969; 22: 309–324.

[3] Emanuel EJ , Emanuel LL . Four models of the physician‐patient relationship. Journal of the American Medical Association, 1992; 267: 2221–2226. 1556799

[4] Szasz TS , Hollnder MH . A contribution to the philosophy of medicine. Archives of Internal Medicine, 1956; 97: 585–592. 13312700 10.1001/archinte.1956.00250230079008

[5] Doron I , Shalev C . From the Patients' Rights Act 1996 to the Dying Patient Act 2005: moving forward or backwards. Journal of Medicine and Law, 2011; 43: 25–34.

[6] Entwistle VA , Carter SM , Cribb A , McCaffery K . Supporting patient autonomy: the importance of clinician‐patient relationships. Journal of General Internal Medicine, 2010; 25: 741–745. 20213206 10.1007/s11606-010-1292-2 PMC2881979

[7] Fallowfield L , Jenkins V . Communicating sad, bad and difficult news in medicine. Lancet, 2004; 363: 312–319. 14751707 10.1016/S0140-6736(03)15392-5

[8] Shaidi J . Not telling the truth: circumstances leading to concealment of diagnosis and prognosis from cancer patients. European Journal of Cancer Care, 2010; 19: 589–593. 20030693 10.1111/j.1365-2354.2009.01100.x

[9] Carpenter B , Dave J . Disclosing a dementia diagnosis: a review of opinion and practice, and a proposed research agenda. Gerontologist, 2004; 44: 149–158. 15075411 10.1093/geront/44.2.149

[10] Johnson H , Bouman WP , Pinner G . On telling the truth in Alzheimer's disease: a pilot study of current practice and attitudes. International Psychogeriatrics/IPA, 2000; 12: 221–229. 10.1017/s1041610200006347 10937542

[11] Ritholz MD , Beverly EA , Brooks KM , Abrahamson MJ , Weinger K . Barriers and facilitators to self‐care communication during medical appointments in the United States for adults with type 2 diabetes. Chronic Illness, 2014; 10: 303–313. 24567195 10.1177/1742395314525647 PMC4157962

[12] Kirk P , Kirk I , Kristjanson LJ . What do patients receiving care for cancer and their families want to be told? British Medical Journal, 2004; 328: 1343–1349. 15151964 10.1136/bmj.38103.423576.55 PMC420285

[13] Swallow VM , Jacoby A . Mothers' evolving relationships with doctors and nurses during the chronic childhood illness trajectory. Journal of Advanced Nursing, 2001; 36: 755–764. 11903705 10.1046/j.1365-2648.2001.02041.x

[14] Berry DL , Wilkie DJ , Thomas CR Jr , Fortner P . Clinicians communicating with patients experiencing cancer pain. Cancer Investigation, 2003; 21: 374–381. 12901283 10.1081/cnv-120018228

[15] Ford S , Fallowfield L , Lewis S . Doctor – patient interactions in oncology. Social Science & Medicine, 1996; 42: 1511–1519. 8771634 10.1016/0277-9536(95)00265-0

[16] Hack TF , Pickles T , Ruether JD , Weir L , Bultz BD , Degner LF . Behind closed doors: systematic analysis of breast cancer consultation communication and predictors of satisfaction with communication. Psycho‐Oncology, 2010; 19: 626–636. 19514095 10.1002/pon.1592

[17] Putnam SM , Stiles WB , Jacob MC , James SA . Teaching the medical interview: an intervention study. Journal of General Internal Medicine, 1988; 3: 38–47. 3339486 10.1007/BF02595755

[18] Cape J , McCulloch Y . Patients' reasons for not presenting emotional problems in general practice consultations. British Journal of General Practice, 1999; 49: 875–879. 10818651 PMC1313556

[19] Knauft E , Nielsen EL , Engelberg RA , Patrick DL , Curtis JR . Barriers and facilitators to end‐of‐life care communication for patients with COPD. Chest, 2005; 127: 2188–2196. 15947336 10.1378/chest.127.6.2188

[20] Hofmann JC , Wenger NS , Davis RB *et al* Patient preferences for communication with physicians about end‐of‐life decisions. Annals of Internal Medicine, 1997; 1: 1–12. 10.7326/0003-4819-127-1-199707010-00001 9214246

[21] Cohen H , Britten N . Who decides about prostate cancer treatment? A qualitative study Family Practice, 2003; 20: 724–729. 14701899 10.1093/fampra/cmg617

[22] Elit L , Charles C , Gold I *et al* Women's perceptions about treatment decision making for ovarian cancer. Gynecologic Oncology, 2003; 88: 89–95. 12586585 10.1016/s0090-8258(02)00090-2

[23] Stevenson FA , Barry CA , Britten N , Barber N , Bradley CP . Doctor‐patient communication about drugs: the evidence for shared decision making. Social Science & Medicine, 2000; 50: 829–840. 10695980 10.1016/s0277-9536(99)00376-7

[24] Carmel S . Behavior, attitudes and expectations regarding the use of life‐sustaining treatments among physicians in Israel: an exploratory study. Social Science & Medicine, 1996; 43: 955–966. 8888465 10.1016/0277-9536(96)00004-4

[25] Carmel S . End‐of‐life care in Israel In: Carmel S, Morse CA, Torres‐Gil FM, Damron‐Rodriguez J, Feldman S, Seedsman T (eds) Lessons on Aging from Three Nations: The Art of Caring for Older Adults. Amityville, NY: Baywood Publishing Company Inc, 2006: 135–152.

[26] Collins DL , Street RL . A dialogic model of conversations about risk: coordinating perceptions and achieving quality decisions in cancer care. Social Science & Medicine, 2009; 68: 1506–1512. 19246146 10.1016/j.socscimed.2009.01.016

[27] Fagerlind H , Lindblad AK , Bergstro I *et al* Patient‐physician communication during oncology consultations. Psycho‐Oncology, 2008; 17: 975–985. 18677715 10.1002/pon.1410

[28] Gordon EJ , Daugherty CK . ‘Hitting you over the head’: oncologists' disclosure of prognosis to advanced cancer patients. Bioethics, 2003; 17: 142–168. 12812182 10.1111/1467-8519.00330

[29] Lamont EB , Christakis NA . Prognostic disclosure to patients with cancer near the end of life. Annals of Internal Medicine, 2001; 134: 1096–1105. 11412049 10.7326/0003-4819-134-12-200106190-00009

[30] Liu WJ , Hu WY , Chiu YF *et al* Factors that influence physicians in providing palliative care in rural communities in Taiwan. Supportive Care in Cancer, 2005; 13: 781–789. 15726431 10.1007/s00520-005-0778-7

[31] Moon PJ . Death‐talks: transformative learning for physicians. The American Journal of Hospice & Palliative Medicine, 2008; 25: 271–277. 18550777 10.1177/1049909108318567

[32] Carmel S . Life‐sustaining treatments: what doctors do, what they want for themselves and what elderly persons want. Social Science & Medicine, 1999; 49: 1401–1408. 10509829 10.1016/s0277-9536(99)00221-x

[33] Moorhead R , Winefield H . Teaching counseling skills to fourth‐year medical students: a dilemma concerning goals. Family Practice, 1991; 8: 343–346. 1800197 10.1093/fampra/8.4.343

[34] Hibbard JH , Stockard J , Mahoney ER , Tusler M . Development of the patient activation measure (PAM): conceptualizing and measuring activation in patients and consumers. Health Services Research, 2004; 39: 1005–1026. 15230939 10.1111/j.1475-6773.2004.00269.x PMC1361049

[35] Holman H , Lorig K . Patients as partners in managing chronic disease: partnership is a prerequisite for effective and efficient health care. British Medical Journal, 2000; 320: 526–527. 10688539 10.1136/bmj.320.7234.526 PMC1117581

[36] Brown RF , Hill C , Burant CJ , Siminoff LA . Satisfaction of early breast cancer patients with discussions during initial oncology consultations with a medical oncologist. Psycho‐Oncology, 2009; 18: 42–49. 18484569 10.1002/pon.1376 PMC4839191

[37] Lin HR , Bauer‐Wu SM . Psycho‐spiritual well‐being in patients with advanced cancer: an integrative review of the literature. Journal of Advanced Nursing, 2003; 44: 69–80. 12956671 10.1046/j.1365-2648.2003.02768.x

[38] Schapira L , Butow P , Brown R , Boyle F . Pessimism is no poison. Journal of Clinical Oncology, 2010; 28: 705–707. 19917853 10.1200/JCO.2009.25.0027

[39] Butow P , Dunn S , Tattersall M . Communication with cancer patients: does it matter? Journal of Palliative Care, 1995; 11: 34–38. 8648521

[40] Hack TF , Degner LF , Parker PA . The communication goals and needs of cancer patients: a review. Psycho‐Oncology, 2005; 14: 831–845. 16200519 10.1002/pon.949

[41] Freedman TG . Prescriptions for health providers. From cancer patients. Cancer Nursing, 2003; 26: 323–330. 12886123 10.1097/00002820-200308000-00011

[42] Marshall AA , Smith RC . Physicians' emotional reactions to patients: recognizing and managing counter transference. The American Journal of Gastroenterology, 1995; 90: 4–8. 7801947

[43] Street RL . Physicians' communication and parents' evaluations of pediatric consultations. Medical Care, 1991; 29: 1146–1152. 1943273 10.1097/00005650-199111000-00006

[44] Fleissig A , Glasser B , Lloyd M . Patients need more than written prompts for communication to be successful. British Medical Journal, 2000; 320: 314–315. PMC1117501 10650042

[45] Galassi JP , Schanberg R , Ware WB . The patient reactions assessment: a brief measure of the quality of the patient‐provider medical relationship. Psychological Assessment, 1992; 4: 346–351.

[46] Lerman CE , Brody DS , Caputo C , Smith DG , Lazaro CG , Wolfson HG . Patients' Perceived Involvement in Care Scale: relationship to attitudes about illness and medical care. Journal of Internal Medicine, 1990; 5: 29–33. 10.1007/BF02602306 2299426

[47] Siminoff LA , Grahama GC , Gordon NH . Cancer communication patterns and the influence of patient characteristics: disparities in information‐giving and affective behaviors. Patient Education and Counseling, 2006; 62: 355–360. 16860520 10.1016/j.pec.2006.06.011

[48] Eggly S , Penner LA , Harper FWK , Ruckdeschel JC , Albrecht TL . Information seeking during “bad news” oncology interactions: question asking by patients and their companions. Social Science & Medicine, 2006; 63: 2974–2985. 16962218 10.1016/j.socscimed.2006.07.012

[49] Street RL , Slee C , Kalauokalani DK , Dean DE , Tancredi DJ , Kravitz RL . Improving physician–patient communication about cancer pain with a tailored education‐coaching intervention. Patient Education and Counseling, 2010; 80: 42–47. 19962845 10.1016/j.pec.2009.10.009 PMC2891619

[50] Sanders T , Skevington S . Do bowel cancer patients participate in treatment decision‐making? Findings from a qualitative study. European Journal of Cancer Care, 2003; 12: 166–175. 12787015 10.1046/j.1365-2354.2003.00370.x

[51] Kaplan S , Andek B , Greenfield S , Ogers W , Ware JE . Patient and visit characteristics related to physicians' participatory decision‐making style results from the medical outcomes study. Medical Care, 1995; 33: 1176–1187. 7500658 10.1097/00005650-199512000-00002

[52] Roberts CS , Cox CE , Reintgen DS , Baile WF , Gibertini M . Influence of physician communication on newly diagnosed breast patients' psychological adjustment and decision‐making. Cancer, 1994; 74 (1 Suppl): 336–341. 8004605 10.1002/cncr.2820741319

[53] Takayamaa T , Yamazakia Y , Katsumata N . Relationship between outpatients' perceptions of physicians' communication styles and patients' anxiety levels in a Japanese oncology setting. Social Science & Medicine, 2001; 53: 1335–1350. 11676404 10.1016/s0277-9536(00)00413-5

[54] Von Essen L , Larsson G , Oberg K , Sjoden PO . Satisfaction with care: associations with health‐related quality of life and psychosocial function among Swedish patients with endocrine gastrointestinal tumors. European Journal of Cancer Care, 2002; 11: 91–99. 12099944 10.1046/j.1365-2354.2002.00293.x

[55] Benbassat J , Pilpel D , Tidhar M . Patients' preferences for participation in clinical decision making: a review of published surveys. Behavioral Medicine, 1998; 24: 81–88. 9695899 10.1080/08964289809596384

[56] Singh JA , Sloan JA , Atherton PJ *et al* Preferred roles in treatment decision making among patients with cancer: a pooled analysis of studies using the Control Preferences Scale. The American Journal of Managed Care, 2010; 16: 688–696. 20873956 PMC3020073

[57] Jenkins V , Fallowfield L , Saul J . Information needs of patients with cancer: results from a large study in UK cancer centers. British Journal of Cancer, 2001; 84: 48–51. 11139312 10.1054/bjoc.2000.1573 PMC2363610

[58] Ryan J , Sysko J . The contingency of patient preferences for involvement in health decision making. Health Care Management Review, 2007; 32: 30–36. 17245200 10.1097/00004010-200701000-00005

[59] Carmel S , Lazar A . Telling the bad news: do the elderly want to know their diagnoses and participate in medical decision making? Harefuah, 1997; 133: 505–509. 9451885

[60] Benjamins MR . Religious influences on trust in physicians and the health care system. International Journal of Psychiatry in Medicine, 2006; 36: 69–83. 16927579 10.2190/EKJ2-BCCT-8LT4-K01W

[61] Burger JM , Cooper HM . The desirability of control. Motivation and Emotion, 1979; 3: 381–393.

[62] Thorne S , Hislop TG , Kuo M . Hope and probability: patient perspectives of the meaning of numerical information in cancer communication. Qualitative Health Research, 2006; 16: 318–336. 16449684 10.1177/1049732305285341

[63] Tulsky JA . advance directives importance of communication skills at the end of life. Journal of the American Medical Association, 2005; 294: 359–365. 16030281 10.1001/jama.294.3.359

[64] Carmel S , Werner P , Zeidenberg H . Physicians' and nurses' preferences in using life‐sustaining treatments. Nursing Ethics, 2007; 14: 665–674. 17901176 10.1177/0969733007080208

[65] Streiner DL . Building a better model: an introduction to structural equation modeling. Canadian Journal of Psychiatry, 2006; 51: 317–324. 16986821 10.1177/070674370605100507

[66] Hoelter JW . The analysis of covariance structures: goodness‐of‐fit indices. Sociological Methods & Research, 1983; 11: 325–344.

[67] Hu L , Bentler PM . Cutoff criteria for fit indexes in covariance structure analysis: conventional criteria versus new alternatives. Structural Equation Modeling, 1999; 6: 1–55.

[68] Fallowfield L , Ford S , Lewis S . No news is not good news: information preferences of patients with cancer. Psycho‐Oncology, 1995; 4: 197–202. 11655006 10.1002/pon.2960040305

[69] Oskay‐Özcelik G , Lehmacher W , Könsgen D *et al* Breast cancer patients' expectations in respect of the physician–patient relationship and treatment management results of a survey of 617 patients. Annals of Oncology, 2007; 18: 479–484. 17272832 10.1093/annonc/mdl456

[70] Wass H . Dying the final stage of growth: Issues and challenges In: Sherron RH, Lumsden DB (eds) Introduction to Educational Gerontology. Washington, DC: Hemisphere Publishing Corporation, 1978: 279–304.

[71] Schou I , Ekeberg O , Ruland CM , Karesen R . Do women newly diagnosed with breast cancer and consulting surgeon assess decision‐making equally? Breast, 2002; 11: 434–441. 14965708 10.1054/brst.2002.0454

[72] Wright KB , Frey LR . Communication and cure in an acute cancer center: the effects of patients' willingness to communicate about health, health‐care environment perceptions, and health status on information seeking, participation in care practices, and satisfaction. Health Communication, 2008; 23: 369–379. 18702001 10.1080/10410230802229886

